# Impact of Angiogenic and Cardiovascular Biomarkers for Prediction of Placental Dysfunction in the First Trimester of Pregnancy

**DOI:** 10.3390/biomedicines11051327

**Published:** 2023-04-29

**Authors:** Madalina Nicoleta Nan, Álvaro García-Osuna, Josefina Mora, Cristina Trilla, Assumpta Antonijuan, Vanesa Orantes, Mónica Cruz-Lemini, Francisco Blanco-Vaca, Elisa Llurba

**Affiliations:** 1 Department of Clinical Biochemistry, Hospital de la Santa Creu i Sant Pau, Institut d’Investigació Biomèdica Sant Pau (IIB Sant Pau), 08041 Barcelona, Spain; nicoleta0511@gmail.com (M.N.N.); jmora@santpau.cat (J.M.); aantonijuan@santpau.cat (A.A.); vorantes@santpau.cat (V.O.); fblancova@santpau.cat (F.B.-V.); 2 Department of Biochemistry and Molecular Biology, Facultat de Biociències, Universitat Autònoma de Barcelona, 08193 Barcelona, Spain; 3 Primary Care Interventions to Prevent Maternal and Child Chronic Diseases of Perinatal and Developmental Network (SAMID-RICORS, RD21/0012) and Maternal and Child Health Development Network (SAMID, RD16/0022), Instituto de Salud Carlos III, 28040 Madrid, Spain; ctrilla@santpau.cat (C.T.); mcruzl@santpau.cat (M.C.-L.); ellurba@santpau.cat (E.L.); 4 Department of Obstetrics and Gynecology, Hospital de la Santa Creu i Sant Pau, Universitat Autònoma de Barcelona, Sant Antoni Maria Claret 167, 08025 Barcelona, Spain; 5 Women and Perinatal Health Research Group, Institut d’Investigació Biomèdica Sant Pau (IIB Sant Pau), Sant Quintí 77-79, 08041 Barcelona, Spain; 6 CIBER de Diabetes y Enfermedades Metabólicas Asociadas, Centro de Investigación Biomédica en Red de Diabetes y Enfermedades Metabólicas Asociadas (CIBERDEM), 28029 Madrid, Spain

**Keywords:** angiogenic factors, placental growth factor (PlGF), soluble fms-like tyrosine kinase-1 (sFlt-1), N-terminal pro-brain natriuretic peptide (NT-proBNP), high-sensitivity cardiac troponin T (hs-TnT), uric acid, preeclampsia, small for gestational age, preterm birth

## Abstract

Algorithms for first-trimester prediction of pre-eclampsia usually include maternal risk factors, blood pressure, placental growth factor (PlGF), and uterine artery Doppler pulsatility index. However, these models lack sensitivity for the prediction of late-onset pre-eclampsia and other placental complications of pregnancy, such as small for gestational age infants or preterm birth. The aim of this study was to assess the screening performance of PlGF, soluble fms-like tyrosine kinase-1 (sFlt-1), N-terminal pro-brain natriuretic peptide (NT-proBNP), uric acid, and high-sensitivity cardiac troponin T (hs-TnT) in the prediction of adverse obstetric outcomes related to placental insufficiency. This retrospective case–control study was based on a cohort of 1390 pregnant women, among which 210 presented pre-eclampsia, small for gestational age infants, or preterm birth. Two hundred and eight women with healthy pregnancies were selected as controls. Serum samples were collected between weeks 9 and 13 of gestation, and maternal serum concentrations of PlGF, sFlt-1, NT-proBNP, uric acid, and hs-TnT were measured. Multivariate regression analysis was used to generate predictive models combining maternal factors with the above-mentioned biomarkers. Women with placental dysfunction had lower median concentrations of PlGF (25.77 vs. 32.00 pg/mL; *p* < 0.001), sFlt-1 (1212.0 vs. 1363.5 pg/mL; *p* = 0.001), and NT-proBNP (51.22 vs. 68.71 ng/L; *p* < 0.001) and higher levels of uric acid (193.66 µmol/L vs. 177.40 µmol/L; *p* = 0.001). There was no significant difference between groups regarding the sFlt-1/PlGF ratio. Hs-TnT was not detected in 70% of the maternal serums analyzed. Altered biomarker concentrations increased the risk of the analyzed complications both in univariate and multivariate analyses. The addition of PlGF, sFlt-1, and NT-proBNP to maternal variables improved the prediction of pre-eclampsia, small for gestational age infants, and preterm birth (area under the curve: 0.710, 0.697, 0.727, and 0.697 vs. 0.668, respectively). Reclassification improvement was greater in maternal factors plus the PlGF model and maternal factors plus the NT-p roBNP model (net reclassification index, NRI: 42.2% and 53.5%, respectively). PlGF, sFlt-1, NT-proBNP, and uric acid measurements in the first trimester of pregnancy, combined with maternal factors, can improve the prediction of adverse perinatal outcomes related to placental dysfunction. In addition to PlGF, uric acid and NT-proBNP are two promising predictive biomarkers for placental dysfunction in the first trimester of pregnancy.

## 1. Introduction

Placental dysfunction, caused by abnormal placentation in early pregnancy, can lead to a wide spectrum of obstetric complications, including pre-eclampsia (PE), preterm birth (PTB), small for gestational age (SGA) infants, or stillbirth [1,2]. These adverse outcomes can subsequently increase the risk of lifelong cardiovascular/metabolic disorders for both mother and offspring [3]. An impaired maternal–placental blood supply is unable to provide sufficient nutrition and oxygen for fetal growth, and stresses maternal body systems. The outcomes of placental insufficiency depend on maternal genetics, epigenetics, habits, and chronic diseases [1]. Pregnant women with cardiovascular risk factors are thus more likely to develop PE, and conversely, those who suffer PE during pregnancy are more likely to develop long-term cardiovascular disease [3,4,5].

Several algorithms combining maternal factors and biomarkers are currently used in clinical practice to predict preterm PE in the first trimester of pregnancy [6]. However, these prove insufficiently effective in the detection of term PE. Until now, no cost-effective screening methods have been available in the first trimester for SGA and PTB [6].

Among predictive biomarkers studied for PE and SGA infants are angiogenic and antiangiogenic factors, such as placental growth factor (PlGF), soluble fms-like tyrosine kinase-1 (sFlt-1), and some cardiac biomarkers [7]. PlGF has proved to be especially useful for first-trimester screening of early-onset PE, but the results obtained with sFlt-1 have been contradictory [8,9,10,11,12]. Multiple studies have also shown that N-terminal pro-brain natriuretic peptide (NT-proBNP), a marker for cardiac failure, is a promising PE predictor in the second and third trimesters of pregnancy [13]. Natriuretic peptides aim to exert a cardioprotective function and inhibit cardiac remodeling, being produced by cardiomyocytes in response to myocardial fiber stretching, low blood pressure, and reduced cardiac output [14]. Cardiac troponins, released into the bloodstream after cardiomyocyte damage, are found in high concentrations in pregnant women with hypertension or PE [15]. Finally, uric acid is a risk factor for metabolic syndrome and cardiovascular disease, and high levels in pregnancy have also been associated with severe PE, PTB, and SGA [16,17,18]. Uric acid is produced in the liver from purine-derived nutritional sources, and its production is triggered by the activation of the xanthine oxidase enzyme, whose activity is induced by oxidative stress and cytokines [19].

The aim of this study is to evaluate the ability of PlGF, sFlt-1, NT-proBNP, high-sensitivity cardiac troponin T (hs-TnT), and uric acid in the first trimester of pregnancy to predict the development of adverse obstetric outcomes related to placental dysfunction (PE, SGA infants, and PTB).

## 2. Materials and Methods

### 2.1. Study Population

This was a retrospective nested case–control study conducted at Hospital de la Santa Creu i Sant Pau between 2016 and 2020. Both case and control groups were selected from a large cohort of 1390 women included in a prospective study on placental insufficiency (study registered in ClinicalTrials.gov, NCT04767438).

Maternal and pregnancy characteristics were obtained by individual chart review. Gestational age was calculated according to fetal crown–rump length (CRL) obtained in the 11.0–13.6 week scan. Data from the first-trimester ultrasound measured transabdominally were collected, i.e., gestational age, CRL, and uterine artery pulsatility indices (UAt-PI). Blood pressure (BP) was measured once in one arm (right or left, without distinction), after a 5 min rest with women seated, at the time of the first-trimester ultrasound, according to our current clinical practice. A calibrated Tensoval Duo Control (Hartmann AG, 89522 Heidenheim, Germany) was used. The mean arterial pressure (MAP) was calculated as diastolic BP + (systolic—diastolic BP)/3.

Cases were included if they presented any of the following outcomes: PE, SGA, or PTB. PE was defined as new onset hypertension (systolic BP ≥ 140 mmHg or diastolic BP ≥ 90 mmHg) detected on repeated occasions after 20 weeks of gestation, proteinuria (dipstick urinalysis ≥ 1+ or protein/creatinine ratio ≥ 30 mg/mmol (0.3 mg/mg)), or another maternal organ dysfunction, following the guidelines of the International Society for the Study of Hypertension in Pregnancy [20]. According to the gestational age at time of delivery, PE was classified into preterm (<37 weeks) and term (≥37 weeks) [6,20]. Small for gestational age (SGA) infant was defined as a birth weight below the 10th centile according to local standards [21,22]. Spontaneous PTB was defined as delivery occurring before 37 weeks of pregnancy [23], in the absence of other maternal or fetal comorbidities. A matched control group was also selected, with matching parameters including maternal age, ethnicity, body mass index (BMI), and gestational age at the time of sampling. Exclusion criteria were major anatomic malformations, confirmed chromosomal or genetic abnormalities, or second-trimester pregnancy loss.

### 2.2. Sample Analysis

Blood samples were obtained along with the routine analysis performed in the first trimester of pregnancy, between weeks 9 and 13, for screening of aneuploidy and PE. Whole blood samples were collected by venipuncture in Vacutainer™ tubes (Becton Dickinson, NJ, USA) and fractionated by centrifugation at 3000 g for 15 min at room temperature to obtain serum, which was aliquoted and stored at −80 °C until analyzed.

Serum concentrations of PlGF, sFlt-1, NT-proBNP, and hs-TnT were measured using automated electrochemiluminescence immunoassays on the Roche Cobas^®^ e601 platform (Roche Diagnostics GmbH, Mannheim, Germany). The serum concentration of uric acid was measured using an automated colorimetric uricase method on the Abbott Alinity^®^ c platform (Abbott Laboratories, Chicago, IL, USA). The measuring ranges were 3–10,000 pg/mL for PlGF, 10–85,000 pg/mL for sFlt-1, 10–35,000 ng/L for NT-proBNP, 3–10,000 ng/L for hs-TnT, and 60–1950 μmol/L for uric acid. Intra-and inter-assay coefficients of variation, evaluated with PreciControl Multimarkers 1 and 2 (Roche Diagnostics) for PlGF and sFlt-1, with PreciControl Cardiac 1 and 2 (Roche Diagnostics) for NT-proBNP and hs-TnT, and with Multichem S Plus 1, 2, and 3 (Technopath Clinical Diagnostics, Ballina, Ireland) for uric acid were found to be <5% in all assays. Concentrations below the limit of detection were expressed as an absolute value of the limit of detection.

### 2.3. Statistical Analyses

Normality of continuous variables was assessed by the Kolmogorov–Smirnov test. A Student’s *t*-test and a Mann–Whitney test were used for parameters following a normal and a non-normal distribution, respectively. Data are presented as mean ± standard deviation (SD) for continuous variables and median (interquartile range (IQR)) for non-continuous variables. Categorical variables were expressed as absolute values and percentages, and they were compared using the Chi-square test.

Biomarker performance for the discrimination of placental dysfunction was assessed by receiver operation characteristic (ROC) curves. Different predictive models combining maternal risk factors and biomarkers were generated using multivariate analysis. Area under the curve (AUC) was determined for each predictive model, which was compared using the DeLong test. For each of the models, sensitivity, specificity, positive predictive value (PPV), negative predictive value (NPV), positive likelihood ratio (LR+), and negative likelihood ratio (LR−) were calculated, and cut-off was defined by Younden method. Based on the most updated expert recommendations, the power of the biomarkers to increase outcome prediction was evaluated by calibration (Hosmer–Lemeshow test), discrimination (integrated discrimination index (IDI)) and reclassification analyses (free net reclassification index (NRI) [24,25]. IDI indicates whether adding a new risk factor to a predictive model improves the prediction. NRI is used to quantify the number of patients that are correctly reclassified by adding a new variable to a predictive model. The category-free NRI was calculated as the sum of the “event NRI” (NRIe) and the “nonevent NRI” (NRIne).

Analyses were performed with SPSS (IBM SPSS Statistics for Windows, Version 25.0. Armonk, NY, USA: IBM Corp) and R (www.R-project.org, version 4.2.3 accessed on 8 April 2023). *p* values below 0.05 were considered statistically significant.

## 3. Results

From the original prospective study cohort of 1390 women with singleton pregnancies, we included 210 of them (15.1%) who developed complications associated with placental dysfunction (cases). Then, a matching control group of 208 was selected. The following diagnoses were confirmed: preterm PE (*n* = 13; 0.9%), term PE (*n* = 38; 2.7%), SGA infants without PE (SGA) (*n* = 126; 9.0%), and spontaneous PTB (*n* = 33; 2.4%).

Maternal and pregnancy characteristics are shown in Table 1. Maternal medical history of chronic pathologies did not differ between case and control groups. The proportion of the following maternal risk factors was higher in the case group: smoking habit, previous PE, previous SGA, conception through assisted reproductive technologies, and MAP. Caucasian ethnicity was higher in the cases group (72.4% vs. 63.9%), with Latin American ethnicity higher in the control group (26.4% vs. 18.1%).

Correlations of the study variables with maternal and pregnancy characteristics at inclusion are shown in Supplementary Table S1. Biomarker concentrations did not correlate with maternal age. sFlt-1, uric acid, and NT-proBNP correlated with BMI. PlGF correlates with gestational age at blood sampling and MAP. hs-TnT was excluded from the analysis, since more than half of the patients studied (70%) had hs-TnT concentrations below the detection limit of the assay (<3 ng/L).

### Biomarker Performance

Table S2 presents the performance of maternal characteristics for the prediction of placental dysfunction; as expected, MAP, history of PE, history of SGA, and smoking were significantly associated with placental complications. Concentrations of PlGF, sFlt-1, and NT-proBNP were lower and uric acid higher in cases when compared to controls, all differences being significant (Table 2). The ability to discriminate between the two groups was significant for all biomarkers. The AUC of NT-proBNP (0.649) was very similar to that of PlGF (0.612). The best cut-off values for the studied biomarkers are shown in Table 2. These cut-offs were used to perform logistic regression models along with maternal factors. AUC for each of the biomarkers and for each outcome (PE, SGA, and PTB) were also calculated and are shown in Table S3. All individually analyzed biomarkers discriminate between women who developed pre-eclampsia and women who did not develop pregnancy complications. PlGF, sFlt-1, and NT-proBNP are able to discriminate between women who developed SGA and those who did not. PlGF, uric acid, and NT-proBNP have a significant AUC discriminating between the PTB group and controls.

In order to combine biomarkers and maternal factors, linear regression models were performed. All variables listed in Table 1 that differed between groups or showed a non-significant trend, and all biomarkers screened in the first trimester of pregnancy, were introduced, and a stepwise linear regression was used to generate predictive models of placental complications. The resulting model with maternal factors includes smoking, previous SGA, assisted reproductive technologies (ART), MAP, and mean UAt-PI. Table 3 shows the odds ratios for all significant variables in the six models. The same analysis was performed for each outcome considering biomarkers and maternal variables that showed significant differences between the control group and women who developed PE, SGA, or PTB (Table S4). In combination with maternal factors, all biomarkers are able to improve pre-eclampsia prediction; however, only PlGF, sFlt-1, and NT-proBNP can improve SGA prediction. Although for the PTB prediction, any maternal factor is significant, women with low concentrations of PlGF and NT-proBNP and high concentrations of uric acid have a higher risk of developing PTB.

To evaluate the incremental usefulness of PlGF, sFlt-1, NT-proBNP, and uric acid, discrimination, calibration, and reclassification tests were performed.

**Figure 1:**
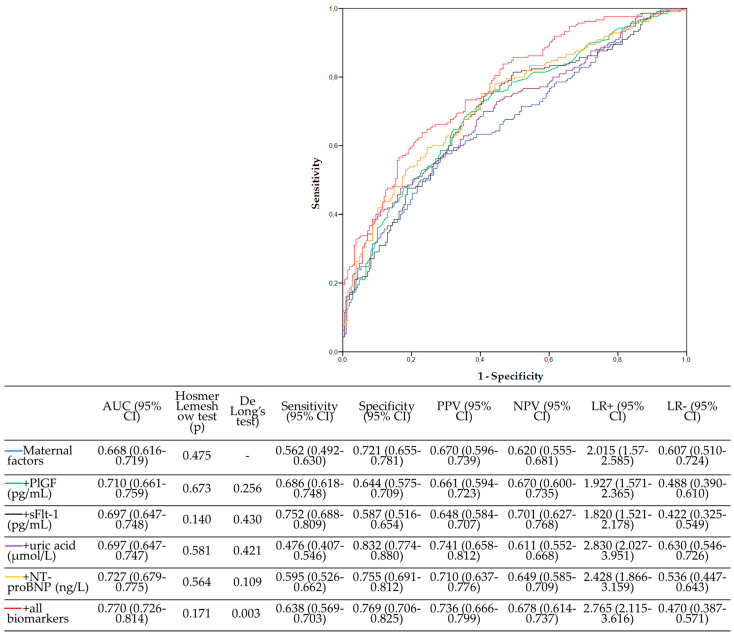
Prediction models for placental dysfunction in the first trimester of pregnancy. All models had the Hosmer–Lemeshow test with *p* > 0.05, confirming their capacity to explain the observed outcomes. PlGF, placental growth factor; sFlt-1, soluble fms-like tyrosine kinase-1; NT-proBNP, N-terminal pro-brain natriuretic peptide; AUC, area under the curve; 95% CI, 95% confidence interval, PPV, positive predictive value; NPV, negative predictive value; LR+, positive likelihood ratio, LR−, negative likelihood ratio.

The ability of the different models to predict pregnancy complications is summarized in Figure 1. Biomarkers were better predictors than maternal risk factors alone, but the improvement was significant only with the combination of all (De Long’s test *p* = 0.003). NPV, PPV, LR+, and LR− are estimated for all models and displayed in Figure 1.

The addition of all the biomarkers improved discrimination between groups in all the models (Table 4). Reclassification improvement was great in the maternal factors plus all biomarkers (NRI > 60%) and intermediate improvement in the other models (NRI around 40%).

To more objectively assess which would be the best biomarker, these were ordered according to their AUC, IDI, and NRI, with the best biomarker valued at 1 and the worst biomarker valued at 5. The ∆AUC is the difference between the AUC of the maternal factors + biomarker model and the maternal factor model. Then, the three scores were added, and the lowest score represented the best model. On a scale of 3 to 14, the best model achieved a sum rank of 3, this being the maternal factor + all biomarkers model. The best individual marker was NT-proBNP, with a sum rank of 6 (Table 4).

## 4. Discussion

The main finding of this study, using a statistical analysis based on the latest expert recommendations, show that concentration of PlGF, sFlt-1, NT-proBNP, and uric acid can predict the development of adverse obstetric outcomes related to placental dysfunction (PE, SGA infants, and PTB) in individual analysis and when combined with maternal factors. Furthermore, this study shows that the addition of sFlt-1 and NT-proBNP to current algorithm models for the prediction of PE in the first trimester of pregnancy improves the performance of screening and, potentially, the ability to identify women destined to develop other placental complications such as GA and PTB.

All screened biomarkers differed between the case and control groups. In accordance with previous studies [8,9], PlGF levels were lower in the case group. This biomarker was one of the best predictors of placental dysfunction, both in the univariate analysis (AUC = 0.612) and multivariate analysis, combined with maternal factors (AUC = 0.710). NT-proBNP concentrations were also lower in pregnant women with placental dysfunction, as described in another study regarding this biomarker in the first trimester [11]. This result contrasts with a large part of studies performed in the second and third trimesters that show an increase in NT-proBNP concentrations associated with a higher incidence of PE [13,26]. However, there are studies reporting that low NT-proBNP levels in the general population are associated with stage 1 hypertension, possibly due to genetic susceptibility [14]. This is supported by the present study, since patients with low NT-proBNP in the first trimester were more likely to develop complications in pregnancy. This decrease in NT-proBNP concentrations could lead to the loss of the cardioprotective effect of natriuretic peptides at the onset of the obstetric complication and would raise the risk of developing these outcomes while in already established PE, the peptide increases due to direct myocardial distension and injury. The ability of NT-proBNP to predict problematic pregnancies was slightly higher compared to PlGF in the univariate analysis (AUC = 0.649) and in the multivariate analysis (AUC = 0.727).

The concentrations of sFlt-1 in the first trimester decreased in the cases with respect to controls, unlike in the second and third trimesters when high sFlt-1 levels are predictive of PE and SGA. Nevertheless, as this biomarker remains stable until week 20, when the concentration begins to increase [27], the levels in the first trimester may be similar to those of the general population. sFlt-1 had less power to predict complications compared with PlGF, both in the univariate (AUC = 0.598) and multivariate (AUC = 0.697) analysis.

Increased uric acid concentrations in the third trimester of pregnancy have been related to the development of PE and adverse outcomes such as PTB and SGA [17,18]. In the present study, we found increased uric acid levels in the cases group. This contrasts with another study where the authors reported unaltered levels in the first trimester [27]. The ability of this biomarker to distinguish between groups was similar to that of sFlt-1, both in the univariate (AUC = 0.596) and multivariate (AUC = 0.697) analysis. These data show that sFlt-1 and uric acid are weak biomarkers for placental dysfunction in the first trimester of pregnancy.

Comparing the different developed models, the predictive capacity of the maternal factors (smoking, previous SGA, ART, MAP, and median UAt-PI) was significantly improved only when combined with all the biomarkers (*p* = 0.001). Models combining clinical factors with PlGF or NT-proBNP did not differ significantly from maternal factors alone (*p* = 0.256, *p* = 0.109). This may be due to the small size of the study population, specifically the small numbers of the outcomes evaluated. Improvements in reclassification by the different models, quantified by the NRI and IDI, were higher when NT-proBNP was included. Moreover, when the rank sum was calculated for biomarker performance based on all the statistical methods used, NT-proBNP obtained the best score when excluding the model with all the biomarkers. The use of all biomarkers had the best performance; however, this would significantly increase the cost of screening. Prospective studies that include NT-proBNP are needed to confirm the performance on the prediction of different pregnancy complications. Despite the better results of NT-proBNP in this study, evidence from current prospective studies recommends the use of PlGF in first-trimester screening [28]. Adding only one biomarker, PlGF, would reduce the cost of the prediction strategy and might be the best cost-effective option.

Cardiac troponins were excluded from the analysis, as patients studied (70%) had hs-TnT concentrations below the detection limit of the assay (<3 ng/L). In addition, the median hs-TnT values of the case and control groups (3.00 and 3.04 ng/L, respectively) could not distinguish them analytically (CV of the internal controls < 5%). This might be due to the characteristic hemodilution in pregnancy or due to an accidental finding. To the best of our knowledge, no comparable data are available in the literature regarding troponin T levels in the first trimester of pregnancy.

In the biomarker analysis for each individual outcome, PlGF and NT-proBNP proved to be useful for predicting all outcomes (Table S3). Although sFlt1 did not serve to predict PTB, nor uric acid to predict SGA, it is possible that increasing sample sizes would highlight significant differences not previously detected.

An important strength of our study is that it relies on a nested prospective cohort which includes complete data regarding first-trimester variables. All patient clinical data were collected specifically for the purpose of studying placental diseases. Another strength of this study is the time point NT-proBNP, hs-TnT, and uric acid blood analysis, since their measurement in the early first trimester of pregnancy is poorly described in the literature. Additionally, our measurements were performed at earlier time points (between 8 and 13 weeks of gestation) in comparison to previous data that mostly were gathered around 12 weeks of gestation. This would be an advantage in our study since data are obtained before the start of aspirin treatment in the high-risk PE population, thus allowing us to evaluate potential very-early PE prediction. Finally, our study analyzed other pregnancy complications associated with placental dysfunction in addition to PE, such as SGA and PTB.

We also acknowledge some limitations in our study. Obstetric outcomes were analyzed jointly due to the low number of cases for each outcome included in this study. However, much data suggest that both PE and SGA, and PTB have a pathophysiological basis based on a primary placental defect [29,30]. This defect is already present in the first trimester [31], and therefore, we believe that the reported data have potential clinical relevance. This study could thus represent a first step for future research evaluating where there are different biomarkers that could be informative of specific obstetric outcomes. The predictive power of our best model (maternal factors + all biomarkers) of placental dysfunction remains low and is quite similar to what is currently described in the bibliography for late PE, SGA, and PTB. However, our study has a much smaller population than other pregnancy screening studies. The objective of this study is not to replace current screening algorithms but to improve their prediction by adding biomarkers of cardiac dysfunction and angiogenic markers. To demonstrate this improvement in the current screening strategies, it would be necessary to include these biomarkers in the screening algorithms and develop a prospective study.

## 5. Conclusions

PlGF, sFlt-1, NT-proBNP, and uric acid measurements in the first trimester of pregnancy may improve the prediction of outcomes related to placental dysfunction when combined with maternal clinical features, with the use of PlGF and NT-proBNP showing the best performances in terms of prediction of placental complications. Further prospective studies are necessary to validate these findings, especially to determine the use of NT-proBNP in first-trimester screening.

## Figures and Tables

**Table 1:** Maternal and pregnancy characteristics of the study population.

	Controls (*n* = 208)	Cases (*n* = 210)	*p*
Maternal age (years)	34 ± 5	33 ± 5	0.843
GA at blood sampling (weeks)	10.6 ± 1.1	10.7 ± 1.1	0.508
Body mass index (kg/m^2^)	23.0 (21.2–26.6)	23.3 (21.0–27.0)	0.863
MAP (mmHg)	81.7 (76.8–86.5)	84.2 (78.7–91.0)	<0.001
Mean UAt-PI	1.70 (1.38–2.00)	1.77 (1.42–2.19)	0.075
Smoking during pregnancy	11 (5.3%)	27 (12.9%)	0.007
Ethnicity			0.024
Caucasian	133 (63.9%)	152 (72.4%)	
Latin American	55 (26.4%)	38 (18.1%)	
Asian	5 (2.4%)	5 (2.4%)	
Afro-Caribbean	6 (2.9%)	2 (1.0%)	
North African	6 (2.9%)	2 (1.0%)	
Other	3 (1.4%)	11 (5.2%)	
Chronic hypertension	2 (1%)	6 (2.9%)	0.157
Thyroid condition	16 (7.7%)	16 (7.6%)	0.978
Diabetes mellitus	1 (0.5%)	4 (1.9%)	0.181
Autoimmune condition	1 (0.5%)	2 (1.0%)	0.568
Neurologic condition	3 (1.4%)	3 (1.4%)	0.991
Thrombophilia	4 (1.9%)	3 (1.4%)	0.694
Renal disease	1 (0.5%)	1 (0.5%)	0.995
Nulliparous	82 (39.4%)	78 (37.1%)	0.632
Previous PE	3 (1.4%)	14 (6.7%)	0.007
Previous SGA	4 (1.9%)	23 (11%)	<0.001
Previous PTB	3 (1.4%)	9 (4.3%)	0.082
Repeated miscarriage	9 (4.3%)	9 (4.3%)	0.983
ART conception	15 (7.2%)	27 (12.9%)	0.055

Values are expressed as mean ± SD, median (interquartile range), or *n* (%). GA, gestational age; MAP, mean arterial pressure; UAt-PI, uterine artery pulsatility indices; PE, pre-eclampsia; SGA, small for gestational age; PTB, preterm birth; ART, assisted reproductive technologies; *p*-values obtained by Mann–Whitney U test, Student’s *t* test, or Chi-squared test where appropriate.

**Table 2:** Screening performance to detect placental dysfunction according to first-trimester serum biomarkers.

	Controls (*n* = 208)	Cases (*n* = 210)	*p*	AUC (95% CI)	Cut-Off (Sensitivity, Specificity)
PlGF (pg/mL)	32.00 (23.90–42.10)	25.77 (17.80–39.14)	<0.001	0.612 (0.558–0.666)	25.86 (0.712, 0.510)
sFlt1 (pg/mL)	1363.5 (1091.5–1795.5)	1212.0 (937.0–1567.0)	0.001	0.598 (0.544–0.652)	1288 (0.557, 0.620)
sFlt1/PLGF ratio	43 (32–61)	45 (30–66)	0.490	0.520 (0.464–0.575)	-
Uric acid (μmol/L)	177.40 (152.88–199.02)	193.66 (164.16–216.73)	0.001	0.596 (0.542–0.650)	199.2 (0.438, 0.760)
NT-proBNP (ng/L)	68.71 (48.48–99.98)	51.22 (31.05–77.68)	<0.001	0.649 (0.597–0.702)	63.35 (0.662, 0.606)

Values are expressed as median (interquartile range); *p*-values obtained by Mann–Whitney U test. PlGF, placental growth factor; sFlt-1, soluble fms-like tyrosine kinase-1; NT-proBNP, N-terminal pro-brain natriuretic peptide; AUC, area under the curve; 95% CI, 95% confidence interval; *p*-values obtained by Mann–Whitney U test. Cut-off was not calculated because AUC is non-significant.

**Table 3:** Multivariable analysis to predict placental dysfunction according to maternal and pregnancy factors and biochemical markers.

Method of Screening/ Variable	Smoking	Previous PE	Previous SGA	ART Conception	MAP (mmHg)	Median UAt-PI	PlGF (pg/mL)	sFlt-1 (pg/mL)	Uric Acid (μmol/L)	NT-proBNP (ng/L)
Univariable analysis	2.6 (1.3–5.5)	4.9 (1.4–17.2)	6.3 (2.1–18.5)	1.9 (0.9–3.7)	1.0 (1.0–1.1)	1.5 (1.0–2.2)	2.6 (1.7–3.8)	2.1 (1.4–3.0)	2.5 (1.6–3.7)	3.0 (2.0–4.5)
Maternal factors	3.0 (1.4–6.5)	ns	5.9 (1.9–17.8)	2.2 (1.1–4.5)	1.1 (1.0–1.1)	1.8 (1.2–2.7)				
+PlGF (pg/mL)	3.1 (1.4–6.8)	ns	6.5 (2.1–19.9)	2.3 (1.2–4.9)	1.0 (1.0–1.1)	ns	2.9 (1.9–4.5)			
+sFlt-1 (pg/mL)	2.7 (1.2–5.9)	ns	6.2 (2.0–18.9)	2.3 (1.1–4.8)	1.0 (1.0–1.1)	1.6 (1.0–2.4)		1.8 (1.2–2.7)		
+Uric acid (μmol/L)	3.1 (1.4–6.8)	ns	5.1 (1.7–15.6)	2.2 (1.1–4.5)	1.1 (1.0–1.1)	1.8 (1.2–2.7)			2.3 (1.5–3.6)	
+NT-proBNP (ng/L)	3.5 (1.6–7.6)	ns	5.4 (1.7–16.7)	2.4 (1.2–5.1)	1.0 (1.0–1.1)	1.8 (1.2–2.7)				2.9 (1.9–4.5)
+all biomarkers	3.2 (1.4–7.4)	ns	5.4 (1.7–17.0)	2.7 (1.2–5.8)	1.0 (1.0–1.1)	ns	2.6 (1.6–4.2)	1.5 (1.0–2.3)	2.3 (1.4–3.7)	2.8 (1.8–4.4)

Values are expressed as odds ratio (95%CI). Maternal factors include smoking, previous SGA, ART, MAP, and mean UAt-PI. PE, pre-eclampsia; SGA, small for gestational age; ART, assisted reproductive technologies; MAP, mean arterial pressure; UAt-PI, uterine artery pulsatility indices; PlGF, placental growth factor; sFlt-1, soluble fms-like tyrosine kinase-1; NT-proBNP, N-terminal pro-brain natriuretic peptide; ns, non-significant (*p* > 0.05).

**Table 4:** Improvements in model performance after the addition of individual biomarkers to the clinical model using the predefined cut-off values.

Biomarker	ΔAUC (95%CI)	Rank ΔAUC	IDI (%) (95%CI)	Rank IDI	NRI (%) (95%CI)	Rank NRI	Rank Sum
Maternal factors							
+PlGF (pg/mL)	0.042	3	3.7 (1.4–6.0)	3	42.2 (23.6–60.8)	3	9
+sFlt1 (pg/mL)	0.029	4	1.7 (0.5–2.9)	5	35.5 (16.6–54.3)	5	14
+Uric acid (μmol/L)	0.029	4	3.1 (1.4–4.8)	4	39.5 (21.8–57.3)	4	12
+NT-proBNP (ng/L)	0.059	2	5.7 (3.5–7.9)	2	53.5 (35.1–72.0)	2	6
+all biomarkers	0.102	1	12.3 (9.1–15.5)	1	65.2 (47.2–83.2)	1	3

Maternal factors included smoking, previous SGA, ART conception, MAP, and median UAt-PI. Improvements in model performance were assessed by the change in AUC, the IDI, and the NRI. Biomarkers are ranked according to their performance relative to the clinical model. The rank sum was calculated as rank (ΔAUC) + rank (IDI) + rank (NRI). PlGF, placental growth factor; sFlt-1, soluble fms-like tyrosine kinase-1; NT-proBNP, N-terminal pro-brain natriuretic peptide; AUC, area under the curve; IDI, integrated dis-crimination index; NRI, free net reclassification index, ΔAUC = AUC(maternal factors+ biomarker)—AUCmaternal factors.

## Data Availability

The data are unavailable due to privacy restrictions.

## Supplementary Materials

The following supporting information can be downloaded at https://www.mdpi.com/article/10.3390/biomedicines11051327/s1. Table S1. Correlations of the study variables with maternal and pregnancy characteristics at inclusion; Table S2. Maternal characteristics’ performance for prediction of placental dysfunction; Table S3. Screening performance to detect PE, SGA, or PTB according to first-trimester serum markers; Table S4. Multivariable analysis to predict PE, SGA, and PTB according to maternal factors and biochemical markers using the predefined cut-off values.
