# Supplementary material for: Impact of Angiogenic and Cardiovascular Biomarkers for Prediction of Placental Dysfunction in the First Trimester of Pregnancy
Source: Biomedicines. 2023 Apr 29;11(5):1327. doi: 10.3390/biomedicines11051327 (PMC10216012; doi:10.3390/biomedicines11051327)
Supplement: Supplementary file 1 [file biomedicines-11-01327-s001.zip › biomedicines-2337729-supplementary.pdf]

biomedicines

MDPI

Supplementary Material

Impact of angiogenic and cardiovascular biomarkers for prediction of placental dysfunction in the first trimester of pregnancy

Madalina Nicoleta Nan[1,2*], Álvaro García-Osuna[1], Josefina Mora[1-3], Cristina Trilla[3,4], Assumpta Antonijuan[1], Vanesa Orantes[1], Mónica Cruz-Lemini[3-5], Francisco Blanco-Vaca[1,2,6], Elisa Llurba[3-5]

Table S1. Correlations of the study variables with maternal and pregnancy characteristics at inclusion.

	Maternal age (Spearman's r, *p*)	GA at blood sampling (Spearman's r, *p*)	MAP (Spearman's r, *p*)	Body mass index (Spearman's r, *p*)
PlGF, pg/mL	0.026, 0.570	0.455, <0.001	-0.106, 0.031	0.025, 0.609
sFlt1, pg/mL	0.003, 0.955	0.023, 0.638	-0.071, 0.145	-0.109, 0.026
Uric acid, µmol/L	0.001, 0.999	0.060, 0.224	0.045, 0.364	0.178, <0.001
NT-proBNP, ng/L	0.045, 0.359	-0.090, 0.068	-0.160, 0.001	-0.182, <0.001

GA, gestational age; MAP, mean arterial blood pressure; PlGF, placental growth factor; sFlt-1, soluble fms-like tyrosine kinase-1; NT-proBNP, N-terminal pro-brain natriuretic peptide

biomedicines

MDPI

Table S2. Maternal characteristics' performance for prediction of placental dysfunction.

	**Controls (n=208)**	**Cases (n=210)**	***p***	**AUC (95% CI)**
Maternal age (years)	34±5	33±5	0.843	0.537 (0.482-0.592)
Body mass index (kg/m²)	10.6±.1.1	10.6±1.1	0.508	0.505 (0.449-0.560)
MAP (mmHg)	81.7 (76.8-86.5)	84.2 (78.7-91.0)	<0.001	0.599 (0.545-0.653)
Mean UAt-PI	1.70 (1.38-2.00)	1.77 (1.42-2.19)	0.075	0.550 (0.495-0.605)
Smoking during pregnancy	11 (5.3%)	27 (12.9%)	0.007	0.538 (0.483-0.593)
African American women	6 (2.9%)	2 (0.9%)	0.149	0.490 (0.435-0.546)
Previous PE	3 (1.4%)	14 (6.7%)	0.007	0.526 (0.471-0.581)
Previous SGA	4 (1.9%)	23 (11%)	<0.001	0.545 (0.490-0.600)
Previous PTB	3 (1.4%)	9 (4.3%)	0.082	0.514 (0.459-0.570)
ART conception	15 (7.2%)	27 (12.9%)	0.055	0.528 (0.473-0.584)

Values are expressed as mean±SD, median (interquartile range) or n (%). MAP, mean arterial pressure; UAt-PI, uterine artery pulsatility indices; PE, preeclampsia; SGA, small for gestational age; PTB, preterm birth; ART, assisted reproductive technologies; AUC, area under the curve; 95% CI, 95% confidence interval; p-values obtained by Mann-Whitney U test, Student's t test or Chi-squared test where appropriate.

Table S3. Screening performance to detect PE, SGA or PTB according to first-trimester serum markers.

	Controls (n=208)	Cases (n=210)	*p*	AUC (95% CI)	Cut-off (Sensitivity, Specificity)
**PE (n=51)**					
PlGF (pg/mL)	32.00 (23.90-42.10)	23.70 (13.31-37.45)	0.003	0.636 (0.541-0.732)	20.76 (0451, 0.856)
sFlt1 (pg/mL)	1363.5 (1091.5-1795.5)	1158.0 (871.6-1524.0)	0.002	0.640 (0.555-0.725)	1251.0 (0.647, 0.644)
sFlt1/PLGF ratio	43 (32-61)	47 (30-87)	0.293	-	-
Uric acid (µmol/L)	177.40 (152.88-199.02)	204.86 (176.39-234.16)	<0.001	0.664 (0.576-0.752)	202.27 (0.549, 0.774)
NT-proBNP (ng/L)	68.71 (48.48-99.98)	36.80 (23.66-57.33)	<0.001	0.733 (0.650-0.815)	51.71 (0.686, 0.731)
**SGA (n=126)**					
PlGF (pg/mL)	32.00 (23.90-42.10)	26.38 (20.32-39.21)	0.005	0.592 (0.528-0.656)	25.87 (0.492,0.712)
sFlt1 (pg/mL)	1363.5 (1091.5-1795.5)	1270.5 (943.0-1576.0)	0.008	0.587 (0.524-0.650)	1138.5 (0.429, 0.731)
sFlt1/PLGF ratio	43 (32-61)	44 (30-62)	0.928	-	-
Uric acid (µmol/L)	177.40 (152.88-199.02)	183.90 (156.3-208.37)	0.210	-	-
NT-proBNP (ng/L)	68.71 (48.48-99.98)	55.59 (35.95-83.58)	<0.001	0.609 (0.549-0.672)	63.35 (0.611, 0.606)
**PTB (n=33)**					
PlGF (pg/mL)	32.00 (23.90-42.10)	24.52 (15.52-36.15)	0.006	0.641 (0.526-0.755)	28.63 (0.656, 0.625)
sFlt1 (pg/mL)	1363.5 (1091.5-1795.5)	1269.0 (949.1-1730.0)	0.182	-	-
sFlt1/PLGF ratio	43 (32-61)	44 (30-74)	0.466	-	-
Uric acid (µmol/L)	177.40 (152.88-199.02)	208.92 (172.01-240.08)	<0.001	0.702 (0.603-0.800)	199.23 (0.636, 0.760)
NT-proBNP (ng/L)	68.71 (48.48-99.98)	45.89 (34.84-67.05)	0.001	0.666 (0.568-0.765)	62.07 (0.688, 0.611)

Values are expressed as median (interquartile range); PE, preeclampsia; SGA, small for gestational age; PTB, preterm birth; PlGF, placental growth factor; sFlt-1, soluble fms-like tyrosine kinase-1; NT-proBNP, N-terminal pro-brain natriuretic peptide; AUC, area under the curve; 95% CI, 95% confidence interval; p-values obtained by Mann-Whitney U test, - cut-off was not calculated because AUC is non-significant.

Table S4. Multivariable analysis to predict PE, SGA and PTB according to maternal factors and biochemical markers using the pre-defined cut-off values.

	Body mass index (kg/m^2)	MAP (mmHg)	Mean UAt-PI	Smoking during pregnancy	Previous PE	Previous SGA	Previous PTB	ART conception	PlGF (pg/mL)	sFlt1 (pg/mL)	Uric acid (μmol/L)	NT-proBNP (ng/L)
**PE**												
Maternal factors	1.1 (1.0-1.2)	1.1 (1.1-1.2)	3.0 (1.4-6.1)		8.6 (1.8-40.1)	ns	ns	3.2 (1.0-9.9)				
+ PlGF	1.1 (1.0-1.2)	1.1 (1.1-1.2)	2.6 (1.2-5.4)		6.8 (1.5-31.0)	ns	ns	3.3 (1.0-10.6)	2.9 (1.3-6.5)			
+ sFlt1	1.1 (1.0-1.2)	1.1 (1.1-1.2)	2.3 (1.1-4.9)		9.9 (2.0-49.1)	ns	ns	3.4 (1.1-10.5)		2.6 (1.2-5.7)		
+ Acid uric	1.1 (1.0-1.2)	1.2 (1.1-1.2)	2.6 (1.2-5.4)		5.9 (1.3-27.4)	ns	ns				1.1 (1.9-8.9)	
+ NT-proBNP	ns	1.1 (1.1-1.2)	2.5 (1.2-5.5)		8.5 (1.7-42.3)	ns	ns	3.5 (1.0-12.2)				5.2 (2.4-11.5)
+ all biomarkers	ns	1.1 (1.1-1.2)	ns		11.6 (2.1-66.7)	ns	ns	4.1 (1.2-14.4)	2.8 (1.1-7.3)	2.9 (1.3-6.7)	3.7 (1.6-8.4)	3.8 (1.7-8.7)
**SGA**												
Maternal factors	0.9 (0.8-0.9)			3.1 (1.4-6.9)		7.8 (2.5-14.1)						
+ PlGF	ns			3.6 (1.6-8.0)		9.0 (2.9-28.3)			2.8 (1.7-4.5)			
+ sFlt1	0.9 (0.8-0.9)			3.1 (1.4-7.1)		7.9 (2.5-24.8)				2.2 (1.3-3.5)		
+ NT-proBNP	0.9 (0.8-0.9)			3.5 (1.5-7.9)		7.0 (2.2-22.3)						2.6 (1.6-4.3)
+ all biomarkers	ns			4.0 (1.7-9.2)		8.0 (2.5-25.7)			3.0 (1.8-4.9)	ns	ns	2.6 (1.6-4.3)
**PTB**												
PlGF									3.3 (1.5-7.2)			
Acid uric											5.5 (2.5-12.0)	
NT-proBNP												3.6 (1.6-7.9)
all biomarkers									3.5 (1.5-8.0)	ns	5.0 (2.2-11.4)	3.4 (1.5-8.0)

To obtain the final model, all the maternal variables that were significantly different between the two groups were introduced and a stepwise linear regression was performed to get the final model of maternal factors for each outcome. Maternal factors for PE include: body mass index, MAP, median UAt-PI, previous PE and ART conception. Maternal factors for SGA include: body mass index, smoking during pregnancy and previous SGA. Any maternal factor is not included in the stepwise linear regression. Values are expressed as odds ratio (95%CI). PE, preeclampsia; SGA, small for gestational age; PTB, premature birth; ART, assisted reproductive technologies; MAP, mean arterial pressure; UAt-PI, uterine artery

pulsatility indices; GA, gestational age; PlGF, placental growth factor; sFlt-1, soluble fms-like tyrosine kinase-1; NT-proBNP, N-terminal pro-brain natriuretic peptide ns, non-significant ($p>0.05$).
